# Adolescent health in rural Ghana: A cross-sectional study on the co-occurrence of infectious diseases, malnutrition and cardio-metabolic risk factors

**DOI:** 10.1371/journal.pone.0180436

**Published:** 2017-07-20

**Authors:** Marie Alicke, Justice K. Boakye-Appiah, Inusah Abdul-Jalil, Andrea Henze, Markus van der Giet, Matthias B. Schulze, Florian J. Schweigert, Frank P. Mockenhaupt, George Bedu-Addo, Ina Danquah

**Affiliations:** 1 Institute of Tropical Medicine and International Health, Charité – Universitaetsmedizin Berlin, Berlin, Germany; 2 Komfo Anokye Teaching Hospital, Kwame Nkrumah University of Science and Technology, Kumasi, Ghana; 3 Department of Physiology and Pathophysiology of Nutrition, Institute of Nutrition Science, University of Potsdam, Potsdam, Germany; 4 Department IV – Nephrology, Charité – Universitaetsmedizin Berlin, Berlin, Germany; 5 Department of Molecular Epidemiology, German Institute of Human Nutrition Potsdam-Rehbruecke, Nuthetal, Germany; 6 Institute for Social Medicine, Epidemiology and Health Economics, Charité –Universitaetsmedizin Berlin, Berlin, Germany; International Nutrition Inc, UNITED STATES

## Abstract

In sub-Saharan Africa, infectious diseases and malnutrition constitute the main health problems in children, while adolescents and adults are increasingly facing cardio-metabolic conditions. Among adolescents as the largest population group in this region, we investigated the co-occurrence of infectious diseases, malnutrition and cardio-metabolic risk factors (CRFs), and evaluated demographic, socio-economic and medical risk factors for these entities. In a cross-sectional study among 188 adolescents in rural Ghana, malarial infection, common infectious diseases and Body Mass Index were assessed. We measured ferritin, C-reactive protein, retinol, fasting glucose and blood pressure. Socio-demographic data were documented. We analyzed the proportions (95% confidence interval, CI) and the co-occurrence of infectious diseases (malaria, other common diseases), malnutrition (underweight, stunting, iron deficiency, vitamin A deficiency [VAD]), and CRFs (overweight, obesity, impaired fasting glucose, hypertension). In logistic regression, odds ratios (OR) and 95% CIs were calculated for the associations with socio-demographic factors. In this Ghanaian population (age range, 14.4–15.5 years; males, 50%), the proportions were for infectious diseases 45% (95% CI: 38–52%), for malnutrition 50% (43–57%) and for CRFs 16% (11–21%). Infectious diseases and malnutrition frequently co-existed (28%; 21–34%). Specifically, VAD increased the odds of non-malarial infectious diseases 3-fold (95% CI: 1.03, 10.19). Overlap of CRFs with infectious diseases (6%; 2–9%) or with malnutrition (7%; 3–11%) was also present. Male gender and low socio-economic status increased the odds of infectious diseases and malnutrition, respectively. Malarial infection, chronic malnutrition and VAD remain the predominant health problems among these Ghanaian adolescents. Investigating the relationships with evolving CRFs is warranted.

## Introduction

Infectious diseases and malnutrition still constitute major public health threats in sub-Saharan Africa. In 2015, communicable diseases, protein-energy malnutrition and micronutrient deficiencies ranked among the top 10 causes of disease burden in this region.[1] In Ghana, malaria remains ubiquitous and highly endemic with an annual incidence of 10,000 per 100,000 at risk,[2] and 26% of children aged 11–17 years are underweight.[3] Iron deficiency and vitamin A deficiency are among the most common micronutrient deficiencies in Ghana.[4]

At the same time, metabolic conditions, such as overweight, type 2 diabetes and hypertension are rapidly emerging in sub-Saharan Africa.[1] Among Ghanaian adolescents, the prevalence of overweight *plus* obesity is 3.2% among boys and 10.4% among girls according to age- and sex-specific cut-offs of Body Mass Index (BMI).[5] Type 2 diabetes occurs at 1.3% among young adults (20–29 years).[6] Moreover, one in five Ghanaians (aged 13–39 years) has hypertension defined by age- and sex-specific percentiles.[7]

The epidemiologic transition from infectious diseases and malnutrition to metabolic conditions due to increased life-expectancy and lower birth rates progresses slowly in sub-Saharan Africa.[8] As a consequence, these entities have been reported to co-occur at the country level, within households and even at the individual level. For instance, pooled data from rural West Africa revealed that 5% of women at childbearing age presented with symptoms of micronutrient deficiencies *plus* overweight, and 5% of mother-child pairs showed childhood stunting *plus* maternal overweight.[9] Today, two-thirds of Africa’s population is aged 10–24 years. This population group can enormously contribute to the well-being of African societies.[10] Yet, the health needs of young adults in Africa’s transitional phase have only insufficiently been examined.[11] For instance, factors for type 2 diabetes among Africans remain controversial,[12] and the extend of (mal-)nutrition-related susceptibility to infectious diseases among adolescents is well-described. Therefore, we aimed at investigating among adolescents in rural Ghana i) the proportions of common infectious diseases (malaria, diagnoses and symptoms compatible with another infectious disease), malnutrition (underweight, stunting, iron deficiency, vitamin A deficiency), and CRFs (overweight and obesity, impaired fasting glucose (IFG), hypertension), ii) the co-occurrence of these entities, and iii) demographic, socio-economic and medical risk factors for these entities.

## Materials and methods

### Study design and population

For this cross-sectional study, 201 adolescent boys and girls were consecutively recruited at the Presbyterian Mission Hospital in Agogo, southern Ghana between June and August 2015. Agogo Hospital is a 250-beds healthcare facility serving the Ashanti-Akim North District with a population of around 170,000.[13] Adolescents underwent a health check-up as part of a long-term follow-up on birth outcomes (manuscript in preparation), i.e., they did not present to hospital because of acute symptoms. Inclusion criteria were reaching the age of 15 years in the year of study conduct, informed written consent, absence of pregnancy, and no previous diagnosis of type 1 diabetes.

After an overnight-fast, venous blood was collected into EDTA for malaria diagnosis, for biomarkers of iron status and vitamin A metabolism, and for fasting plasma glucose (FPG). Axillary body temperature (°C), blood pressure (BP) and anthropometric measures were taken by trained study personnel. Socio-demographic data and medical history were documented in questionnaire-based interviews.

The study protocol was reviewed and approved by the Ethics Committee of the Kwame Nkrumah University of Science and Technology, Kumasi. Written informed consent was obtained from all caregivers and assent was given by all participants.

### Physical examinations

All participants underwent a routine clinical examination by the study physician, and current diagnoses were documented. We measured axillary body temperature (°C; bosotherm flex, Bosch + Sohn, Germany) and anthropometric measures were taken in light clothes. Body weight was measured to the nearest 0.5 kg (Camry Person Scale, Model DT602, Hong Kong, China) and height was measured to the nearest 0.1 cm (Seca 213, Hamburg, Germany). Body Mass Index (BMI) was calculated as weight/(height)^2^ in kg/m^2^, and BMI-for-age z-scores (BAZ) and height-for-age z-scores (HAZ) were determined using the software package AnthroPlus (version 1.0.4, World Health Organization [WHO], Geneva, Switzerland). According to the WHO, overweight in adolescent age was defined as 1 ≤ BAZ < 2, obesity as BAZ ≥ 2, underweight (or thinness) as BAZ < -2, and stunting as HAZ < -2.

Systolic and diastolic BP were measured in triplicates every 3 minutes with an automated device (Tel-O-Graph BT, I.E.M. Stolberg, Germany) and appropriate cuffs in a separate room after a minimum of 5 minutes resting time. Mean systolic and mean diastolic BP were calculated using the last two measurements. Hypertension was defined as having a mean systolic or a mean diastolic BP >95^th^ percentile of age-, sex- and height-specific reference data.[14]

### Questionnaire-based interviews

Trained staff conducted questionnaire-based interviews (S1 Questionnaires) to document demographic data (age, sex, ethnic group, residence, place of school) and socio-economic status (SES). Even though the questionnaire had not been validated, it was successfully applied in the same geographic area in a case-control study for risk factors of type 2 diabetes and hypertension.[12] The presence of 11 household assets (electricity, pipe-borne water, radio, TV, fan, cupboard, fridge, bicycle, motorbike, car, cattle) was examined and a wealth-score was calculated as the proportion of present household assets. We recorded literacy of the child, parental education (none, primary, secondary, tertiary), parental occupation (intellectual, manual, other, unemployed), the number of people in the household, and the number of siblings. For medical history, current complaints and fever in the last 48h were documented.

### Laboratory analyses

Laboratory analyses were performed within 4 hours after venous blood collection. Plasma was separated by centrifugation at 8000 rpm for 10 min. Full blood and plasma aliquots were transported to Germany on dry ice and stored at -80°C.

#### Malaria diagnosis

Malaria parasites were counted microscopically on Giemsa-stained thick blood films per 200 white blood cells. Following DNA extraction (QIAamp DNA blood mini kit, Qiagen, Hilden, Germany), semi-nested PCR assays were performed to ascertain *Plasmodium* infection and parasite species.[15] A malarial infection was present, if either microscopy or PCR result was positive. Clinical malaria was defined as positive microscopy for any *Plasmodium* species *plus* current fever (≥37.5°C) or a self-reported history of fever within the last 48h.

#### Biomarkers of malnutrition

For iron status, plasma concentrations of ferritin and C-reactive protein (CRP) were measured by immunoturbidimetry (Architect 16000, ABBOTT Laboratories, Chicago, USA). The inter-assay coefficients of variation were 0.85–2.15% for CRP and 9% for ferritin. Iron deficiency was defined as ferritin < 15 μg/L or as ferritin < 30 μg/L, if CRP was > 0.5 mg/L.[16]

For vitamin A metabolism, retinol concentrations were quantified by high-performance liquid chromatography (HPLC).[17] Vitamin A deficiency was defined according to WHO as a plasma retinol concentration < 0.7 μmol/L.[18]

#### Fasting plasma glucose

For FPG measurement, we used a portable device (Accu-Check Inform II, Roche Diagnostics, Germany). The inter-assay coefficient of variation was 2.9–4.1%. Impaired fasting glucose was defined according to American Diabetes Association criteria as 5.6 mmol/L ≤ FPG ≤ 6.9 mmol/L.[19]

### Statistical analysis

Thirteen participants with missing or implausible values for age, sex, biomarkers or covariates were excluded from the analysis, resulting in a final analytical sample of 188. Infectious diseases were defined as a malarial infection or a diagnosed infectious disease (by study physician) or self-reported symptoms compatible with another infectious disease (e.g. cough, cold, fever); malnutrition comprised underweight, stunting, iron deficiency and vitamin A deficiency; and CRFs were defined as overweight or obesity, IFG or hypertension.

Given an α-level of 0.05, this study had a statistical power of 70% to detect a disease occurrence of 20% ± 7% (e.g. hypertension [7]). For all categorical variables, data are presented as percentage with 95% confidence interval (CI) as a measure of accuracy. Continuous variables are presented as median and interquartile range (IQR). Between-group comparisons were performed by Mann-Whitney-U test for continuous variables and by χ²-test for categorical variables. For the associations of demographic, socio-economic and medical factors with infectious diseases, malnutrition and CRFs, we used logistic regression to calculate odds ratios (OR) and their 95% CIs. Due to the small sample size of our study, we aimed at reducing the number of socio-economic variables for the risk factor analysis. Thus, we investigated the correlation structure of all SES variables using Spearman correlations. Variables with the strongest correlations were selected for further analysis. Therefore, the final regression model for the associations with infectious diseases, malnutrition and CRFs comprised age, sex, residence, maternal occupation, paternal occupation, the wealth score and all other entities. As a sensitivity analysis, we calculated logistic regression models with the same set of risk factors to investigate the relationships within the combined entities.

Statistical analyses were performed by IBM SPSS statistical software package version 23 (IBM, Armonk, NY, USA). The significance threshold was *p* < 0.05.

## Results

### Study population

The demographic and socio-economic characteristics of the study participants are shown in Table 1. The median age was 15.2 years (range: 14.4–15.5 years) and both sexes were equally represented. The majority of adolescents were of Akan ethnicity (93%) and two-thirds lived in Agogo. Most boys and girls attended school (98%) and were able to read and write (90%). These characteristics were similar between male and female participants. Secondary school education predominated among parents, most worked manually. The median number of people in the household was 11, and the median number of siblings was 4 (Table 1).

**Table 1:** Socio-demographic characteristics of 188 rural Ghanaian adolescents.

Characteristic	Male (n = 94)	Female (n = 94)
Age in years	15.2 (15.0–15.4)	15.2 (14.9–15.5)
Ethnic group, Akan (%)	88	97
Residence, Agogo (%)	70	71
Place of school, Agogo	53	64
Wealth score	0.45 (0.29–0.81)	0.55 (0.28–0.82)
Literacy, illiterate (%)	14	5
Education of the father (%)		
None	3	4
Primary	13	11
Secondary	46	46
Tertiary	7	10
Unknown	31	30
Education of the mother (%)		
None	5	3
Primary	16	21
Secondary	42	53
Tertiary	5	1
Unknown	32	21
Occupation of the father (%)		
Intellectual worker	18	30
Manual worker	70	57
Other worker	7	11
Unemployed	4	2
Occupation of the mother (%)		
Intellectual worker	7	7
Manual worker	87	85
Other worker	3	2
Unemployed	2	5
Number of people in the household	11 (2–19)	11 (3–23)
Number of siblings	4 (2–6)	4 (2–6)

Data are presented as median (interquartile range) for continuous variables and as percentage for categorical variables.

### Proportions of infectious diseases, malnutrition and cardio-metabolic risk factors

**Fig 1:**
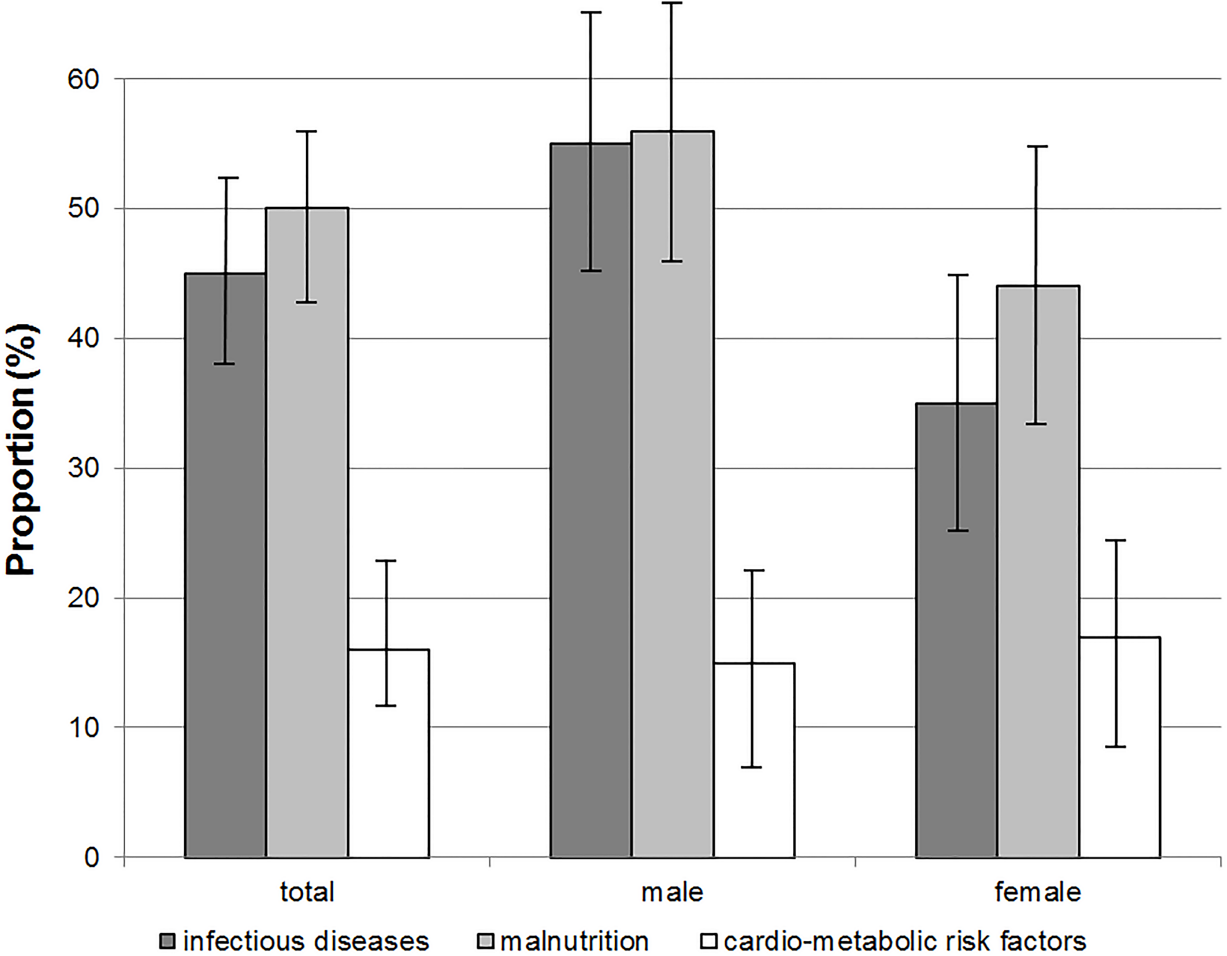
Proportions of infectious diseases, malnutrition and cardio-metabolic risk factors in 188 adolescents in rural Ghana. Error bars indicate 95% confidence intervals. dark grey = infectious diseases, comprise malarial infection *plus* diagnoses of and symptoms compatible with another infectious disease; light grey = malnutrition, comprises underweight, stunting, iron deficiency and vitamin A deficiency; white = cardio-metabolic risk factors, comprise overweight, obesity, impaired fasting glucose and hypertension.

Fig 1 shows the proportions of infectious diseases, malnutrition and CRFs, while Table 2 presents the clinical and anthropometric characteristics. Among the adolescents, 45% (95% CI: 38–52%) had at least one infectious disease, half (95% CI: 43–57%) showed malnutrition, and 16% (95% CI: 11–21%) had at least one CRF. Infectious diseases and malnutrition were more common in boys than in girls, respectively, while overweight/obesity was more common in girls (Fig 1).

**Table 2:** Clinical and anthropometric characteristics of 188 rural Ghanaian adolescents.

Characteristic	Total (n = 188)	Male (n = 94)	Female (n = 94)	*p*
**Infectious diseases**				
Malarial infection (%)				
by microscopy	16 (11, 22)	21 (13, 30)	12 (5, 18)	0.077
by PCR	40 (33, 47)	49 (39, 59)	31 (21, 40)	0.011
by microscopy or PCR	41 (34, 48)	51 (41, 61)	31 (21, 40)	0.005
Geometric mean parasite density (/μL)	160 (46–555)	200 (85–469)	98 (28–340)	0.170
Symptoms/diagnoses for another infectious disease (%)	7 (4, 11)	7 (2, 13)	7 (2, 13)	1.000
History of fever within the last 48h (%)	18 (12, 23)	14 (7, 21)	21 (13, 30)	0.180
**Malnutrition**				
**Macronutrients:**				
Body Mass Index (BMI; kg/m^2^)	18.98 (15.93–22.03)	18.70 (16.0–21.4)	19.37 (16.95–22.79)	0.002
BMI-for-age z-score (BAZ)	-0.43 (-1.63–0.77)	-0.55 (-1.85–0.75)	-0.37 (-1.65–0.91)	0.017
Height-for-age z-score (HAZ)	-0.89 (-2.16–0.38)	-1.17 (-2.57–0.23)	-0.65 (-1.85–0.55)	0.006
Underweight (BAZ ≤ -2, %)	7 (3, 11)	10 (4, 16)	4 (0, 8)	0.151
Stunting (HAZ ≤ -2, %)	15 (10, 20)	21 (13, 30)	9 (3, 14)	0.014
**Micronutrients:**				
Ferritin (μg/L)	57.4 (7.5–107.3)	62.8 (15.1–110.5)	51.6 (5.00–104.4)	0.006
Iron deficiency (ferritin < 15 μg/L or < 30 μg/L, if CRP > 0.5 mg/dL, %)	4 (1, 7)	1 (-1, 3)	7 (2, 13)	0.030
Retinol (μmol/L)	0.77 (0.49–1.05)	0.75 (0.50–1.00)	0.77 (0.50–1.05)	0.231
Vitamin A deficiency (retinol < 0.7 μmol/L, %)	36 (29, 43)	40 (30, 51)	32 (22, 42)	0.225
**Cardio-metabolic risk factors**				
Overweight or obesity (BAZ ≥ 1) (%)	7 (4, 11)	4 (0, 8)	11 (4, 17)	0.096
Fasting plasma glucose (mmol/L)	4.3 (3.5–5.1)	4.3 (3.4–5.2)	4.2 (3.4–5.0)	0.503
Impaired fasting glucose (5.6–6.9 mmol/L) (%)	1 (0, 3)	1 (-1, 3)	1 (-1, 3)	1.000
Mean systolic blood pressure (BP) (mmHg)	110 (95–125)	111 (98–124)	109 (87–125)	0.124
Mean diastolic BP (mmHg)	68 (56–80)	68 (56–80)	68 (56–80)	0.942
Hypertension (BP > 95^th^ percentile or previously diagnosed, %)	9 (4, 13)	10 (4, 16)	7 (2, 13)	0.601

Data are presented as median (interquartile range) for continuous variables and as percentage (95% confidence interval) for categorical variables. Comparisons between males and females were made by Mann-Whitney-U test for continuous variables and by χ^2^-test for categorical variables.

For infectious diseases, 41% of the teenagers presented with malarial infection of generally low parasite density or detected by PCR only (Table 2). *Plasmodium falciparum* was the predominant parasite species (39%; *P*. *ovale*, 17%; *P*. *malariae*, 3%). Malarial infection was more frequent in boys than in girls (*p* = 0.005). Symptomatic malaria was observed in 2% of the juveniles. Current diagnoses or symptoms compatible with another infectious disease were seen in 7% of adolescents with no gender difference (Table 2). Recorded diagnoses were worm infestations, urinary tract infection, *fluor genitalis*, candidiasis, common cold, typhoid fever and chicken pox. Symptoms compatible with another infectious disease comprised cough, cold, white vaginal discharge and fever.

With respect to malnutrition, 7% of study participants were underweight and 15% were stunted. No gender-related differences were observed for underweight, but stunting was more common among boys. The median concentration of CRP was 0.63 mg/L (IQR: 0.10–2.11 mg/L), and this was similar between boys and girls (*p* = 0.32). Iron deficiency was seen in 4% and was more frequent among girls. For vitamin A deficiency, the overall proportion was 36%, with no differences between boys and girls (Table 2).

Regarding CRFs, 7% of adolescents were overweight or obese. This figure was higher in girls than in boys (11% vs. 4%; *p* = 0.096). FPG was normal in most teenagers (4.3 ± 0.6 mmol/L), but IFG was seen in one boy and one girl. The proportion of hypertension was 9% and this was similar between males and females.

### Co-occurrence and risk factors of infectious diseases, malnutrition and cardio-metabolic risk factors

**Fig 2:**
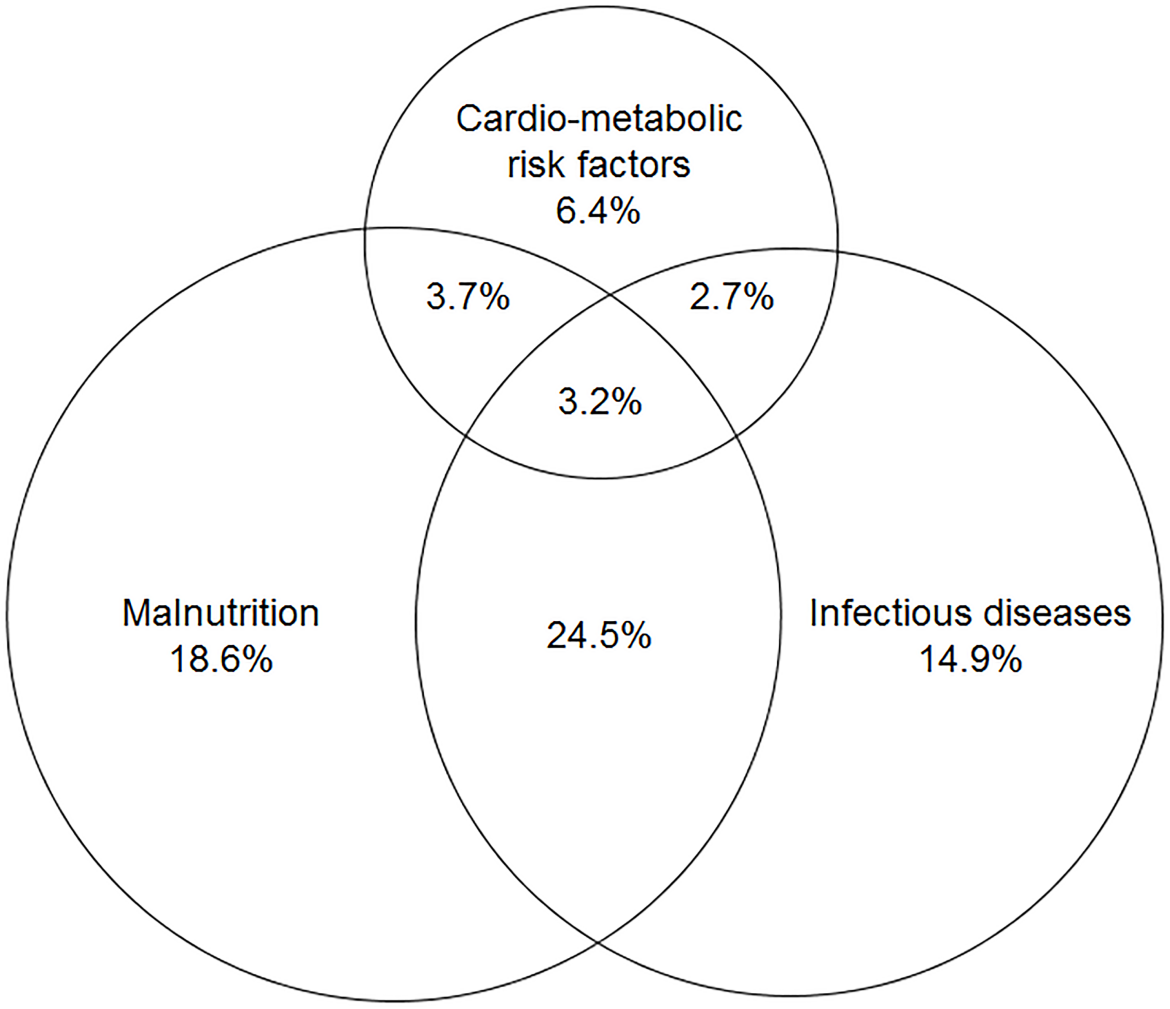
Venn diagram for the co-occurrences of infectious diseases, malnutrition and cardio-metabolic risk factors in 188 adolescents in rural Ghana. Data are presented as proportions of the total study population. Infectious diseases comprise malarial infection *plus* diagnoses of and symptoms compatible with another infectious disease; malnutrition comprises underweight, stunting, iron deficiency and vitamin A deficiency; cardio-metabolic risk factors comprise overweight, obesity, impaired fasting glucose and hypertension.

In Fig 2, we present the co-occurrence of infectious diseases, malnutrition and CRFs. Of all participants, roughly one-third (28%; 95% CI: 21–34%; n = 53) had an infectious disease and concomitant malnutrition. This was dominated by malarial infection *plus* vitamin A deficiency (n = 32/53). Further, the combination of CRFs with either infectious diseases or with malnutrition was discernible in 6% (95% CI: 2–9%; n = 11) and 7% (95% CI: 3–11%; n = 13), respectively. The former mainly comprised malarial infection *plus* hypertension (n = 8/11), while the latter was largely attributable to vitamin A deficiency *plus* overweight or obesity (n = 7/13).

In Table 3, we present crude and multiple-adjusted associations of demographic, socio-economic and medical factors with infectious diseases, malnutrition and CRFs. In univariate analysis, female gender reduced the odds of infectious diseases, while manual paternal occupation (vs. intellectual occupation), a wealth score < median of 0.55 and malnutrition each more than doubled the odds of infectious diseases. In the multivariate model, the associations remained for sex, wealth score and malnutrition (Table 3). Conversely, infectious diseases conferred increased odds of malnutrition. Moreover, male gender and indicators of low SES (parental occupational status) tended to increase the odds of malnutrition in the multivariate model. Regarding CRFs, none of the risk factors was significantly associated. Yet, female gender and low parental occupational status nominally increased the odds of prevalent CRFs.

**Table 3:** Associations of demographic, socio-economic and medical factors with infectious diseases, malnutrition and CRFs.

Risk factors	N	Infectious diseases		Malnutrition		Cardio-metabolic risk factors	
		Crude OR	Multivariate OR	Crude OR	Multivariate OR	Crude OR	Multivariate OR
		(95% CI)	(95% CI)	(95% CI)	(95% CI)	(95% CI)	(95% CI)
Age (per 1 month)		0.93 (0.83, 1.04)	0.91 (0.80, 1.04)	0.93 (0.83, 1.04)	0.95 (0.84, 1.07)	0.91 (0.79, 1.05)	0.91 (0.78, 1.05)
Sex							
Male	94	Reference	Reference	Reference	Reference	Reference	Reference
Female	94	**0.44 (0.24, 0.79)**	**0.54 (0.28, 1.02)**	0.60 (0.34, 1.07)	0.65 (0.35, 1.21)	1.17 (0.54, 2.56)	1.06 (0.46, 2.43)
Residence							
Village	55	Reference	Reference	Reference	Reference	Reference	Reference
Agogo	133	0.72 (0.39, 1.36)	0.61 (0.30, 1.26)	0.77 (0.41, 1.45)	0.88 (0.44, 1.76)	0.80 (0.35, 1.83)	0.80 (0.33, 1.96)
Occupation of the father							
Intellectual worker	45	Reference	Reference	Reference	Reference	Reference	Reference
Manual worker	120	**2.14 (1.05, 4.37)**	1.06 (0.45, 2.50)	1.77 (0.88, 2.26)	1.76 (0.77, 4.00)	1.15 (0.42, 3.12)	1.39 (0.45, 4.34)
Other worker	17	1.40 (0.44, 4.41)	0.60 (0.15, 2.36)	1.33 (0.43, 4.10)	1.33 (0.35, 5.04)	2.71 (0.70, 10.47)	2.57 (0.55, 12.12)
Unemployed	6	0.40 (0.04, 3.74)	0.23 (0.02, 2.53)	1.50 (0.27, 8.28)	1.77 (0.28, 11.18)	1.30 (0.13, 13.13)	1.75 (0.15, 19.84)
Occupation of the mother							
Intellectual worker	14	Reference	Reference	Reference	Reference	Reference	Reference
Manual worker	162	2.27 (0.68, 7.52)	2.04 (0.54, 7.71)	1.76 (0.56, 5.47)	1.33 (0.39, 4.59)	1.10 (0.23, 5.19)	1.08 (0.20, 5.79)
Other worker	5	3.75 (0.45, 31.62)	3.32 (0.28, 39.41)	7.20 (0.62, 83.34)	5.47 (0.40, 74.69)	4.00 (0.39, 41.23)	2.89 (0.21, 39.34)
Unemployed	7	0.42 (0.04, 4.66)	0.22 (0.02, 3.13)	4.50 (0.63, 32.30)	5.39 (0.68, 42.51)	1.00 (0.08, 13.37)	1.00 (0.07, 14.92)
Wealth score							
≥ median (0.55)	101	Reference	Reference	Reference	Reference	Reference	Reference
< median (0.55)	87	**2.33 (1.29, 4.19)**	**2.60 (1.30, 5.21)**	0.88 (0.50, 1.56)	0.56 (0.29, 1.12)	0.74 (0.33, 1.63)	0.71 (0.29, 1.71)
Infectious disease							
Negative	103	-	-	Reference	Reference	Reference	Reference
Positive	85	-	-	**2.29 (1.27, 4.12)**	**2.26 (1.18, 4.33)**	0.66 (0.29, 1.47)	0.68 (0.29, 1.64)
Malnutrition							
Negative	94	Reference	Reference	-	-	Reference	Reference
Positive	94	**2.29 (1.27, 4.12)**	**2.27 (1.18, 4.34)**	-	-	0.73 (0.33, 1.60)	0.67 (0.29, 1.58)
Metabolic condition							
Negative	158	Reference	Reference	Reference	Reference	-	-
Positive	30	0.66 (0.29, 1.47)	0.69 (0.28, 1.69)	0.73 (0.33, 1.60)	0.67 (0.29, 1.59)	-	-

Odds ratios (OR) and 95% confidence intervals (CIs) were calculated by logistic regression; multivariate models include all other variables. Infectious diseases comprise malarial infection *plus* diagnoses of and symptoms compatible with another infectious disease; malnutrition comprises underweight, stunting, iron deficiency and vitamin A deficiency; cardio-metabolic risk factors comprise overweight, obesity, impaired fasting glucose and hypertension.

As a sensitivity analysis, we used the same set of demographic and socio-economic variables to calculate i) associations between malarial infection or other infectious diseases and nutrient deficiencies, ii) relationships between macro- and micronutrient deficiencies, and iii) interrelations between overweight/obesity and IFG or hypertension. Neither malarial infection nor other common infectious diseases were associated with underweight, stunting or iron deficiency. The presence of vitamin A deficiency increased the odds for common infectious diseases (OR: 3.23; 95% CI: 1.03, 10.19), but not for malarial infection. Also, anthropometric markers of protein-energy-malnutrition, i.e. underweight or stunting, had no effect on micronutrient deficiencies (iron, vitamin A). For IFG, the occurrence was too low (n = 2) to calculate regression models, while there was a lack of association between overweight/obesity and hypertension (OR: 0.79; 95% CI: 0.09, 6.72).

## Discussion

### Summary of main findings

In rural Ghana, we investigated the proportions and the co-occurrence of common infectious diseases, malnutrition and CRFs among 188 adolescents. Demographic, socio-economic and medical risk factors for the combined entities were assessed as well as associations of single diseases within these entities. Roughly, half of the study population had an infectious disease or was malnourished; 16% presented with a CRF. Infectious diseases and malnutrition were more common among boys, while CRFs tended to be more frequent among girls. Infectious diseases and malnutrition were strongly linked with each other (co-occurrence 28%). Particularly, vitamin A deficiency increased the risk of non-malarial infectious diseases more than 3-fold. Moreover, male gender and low household SES increased the odds of both, infectious diseases and malnutrition. The overlap of infectious diseases and malnutrition with CRFs was rather small (2 out of 25 teenagers), and no associations of demographic, socio-economic and medical factors with CRFs were observed.

### Proportions of infectious diseases, malnutrition and cardio-metabolic risk factors

For malarial infection, there is a marked paucity of prevalence data from the adolescent population in Ghana. Compared to younger age groups in the country [20], we found a lower proportion of *Plasmodium* infections (41%) which were largely asymptomatic and of low or submicroscopic parasite density, arguing for a naturally acquired semi-immunity among these Ghanaian teenagers.[21] In general, the study findings may not be representative for Ghanaian adolescents, because of the limited sample size. Moreover, we focused on malaria and infectious diseases that are common and readily detectable. Thus, we might have underestimated the proportions of other common infectious diseases requiring diagnostic tests beyond routine physical examination, such as HIV/AIDS, tuberculosis and so-called neglected tropical diseases.[2]

The present estimates for malnutrition contribute uniquely to the scarce data of the teenage group in Ghana. Regarding macronutrient deficiencies, our findings suggest somewhat lower figures than expected for underweight (7%) [3], and similar proportions for stunting (15%).[22] At the same time, the male preponderance of macronutrient deficiencies has been frequently reported from SSA [3, 22] and is attributed to higher levels of physical activity due to manual labour among boys.[23] For micronutrient deficits, the degree of iron deficiency (4%) in the present study population was lower than previously reported [24], whereas the proportion of vitamin A deficiency (36%) was similar.[25]

For CRFs, the proportion (7%) and the female preponderance of overweight/obesity accord with previous reports from the region.[3, 12, 26] Likely, the differences in study design and in the degree of urbanization contribute to the comparatively low proportion of IFG in the present analysis.[27, 28] With regard to hypertension, the proportion of 9% was surprisingly high, compared to previous reports from urban Ghana (4%) [29], and given the percentile-based definition of hypertension (expected prevalence: 5%). While the available reference data stem from a large multi-ethnic survey [14], their application in sub-Saharan African settings is novel and may require independent verification.

### Co-occurrence and risk factors of infectious diseases, malnutrition and metabolic conditions

The vicious circle of infectious diseases and malnutrition remains a major public health challenge in sub-Saharan Africa.[1] This seems to apply to adolescents in rural Ghana, too. Almost one-third of our study population presented with an infectious disease *plus* at least one form of nutritional deficits. Malarial infection and vitamin A deficiency were the predominant conditions (32/53). The association between clinical malaria and malnutrition has extensively been examined [30], and is seen also for asymptomatic infections among adolescents elsewhere in sub-Saharan Africa.[31] In our study, malarial infection and other common infectious diseases increased the odds of malnutrition 2.3-fold; and this was also observed *vice versa*. Moreover, low occupational status of the father and low wealth score increased the odds of infectious diseases and of malnutrition, in accordance with current findings from sub-Saharan Africa, linking poverty and disease.[1] More specifically, vitamin A deficiency was strongly associated with common infectious diseases (other than malaria), a finding that is commonly attributed to impaired mucosal epithelial regeneration and immune dysfunction.[32] Despite a considerable reduction of vitamin A deficiency-associated diseases in West Africa in the past 20 years, our results underscore that vitamin A deficiency remains the fourth leading cause of disease burden in this region.[33]

On the background of demographic and economic development, Ghana faces an epidemiologic transition from infectious diseases to metabolic conditions that appears to be delayed in rural areas and poorer social classes.[34, 35] Consequently, infectious diseases still predominate while metabolic conditions increase steadily. This “double burden of disease” has been recognized on the country level,[35] but only selective efforts were made to re-conceptualize healthy body ideals and to improve health literacy in Ghana.[36, 37] In the present study, we assessed the co-occurrence of infectious diseases and CRFs in the individual. This proportion of 6% was dominated by malarial infection *plus* hypertension. It appears unlikely that high blood pressure was an immediate consequence of malarial infection, as indicated by the lack of association in our study. Rather, malarial infection and malaria-related fever reduce systolic blood pressure [38], and our observations probably reflect paralleling diseases.

While the term “double burden of malnutrition” usually refers to the co-occurrence of underweight, stunting or micronutrient deficiencies *plus* overweight or obesity, the denominator for this constellation frequently varies. On the country level, the Double burden of malnutrition refers to considerable amounts of childhood stunting (27%) and maternal overweight (29%) in the Ghanaian population.[4] On the household level, the term describes families with at least one underweight, stunted or micronutrient deficient member *plus* at least one overweight or obese person.[39] For the individual level, the double burden of malnutrition addresses macro- and micronutrient deficiencies as comorbidities of adiposity in one person. The present study extends the latter concept to the co-occurrence of nutritional deficits *plus* overweight, obesity, IFG and hypertension. A similar analysis was conducted among urban adults aged 25–60 years in Burkina Faso, and revealed that one-quarter of the study population had at least one nutritional deficiency and one CRF (overweight or obesity or abdominal obesity, hypertension, hyperglycaemia or insulin resistance or diagnosed diabetes and dyslipidaemia).[40] Already at the age of 15 years, nutritient deficiencies *plus* CRFs were present in 7% of our study population, which was mainly attributed to vitamin A deficiency *plus* hypertension. Hypertension rates in Ghana are projected to increase dramatically, based on population growth and aging [41], while vitamin A deficiency still manifests in 2% of women at childbearing age.[42] Therefore, once the adolescents get older, the group of vitamin A deficient and hypertensive adults will definitely grow, challenging diagnosis and management of these entities.[43]

### Strengths and limitations

So far, data on the co-occurrence of infectious diseases, malnutrition and CRFs are scarce for the population group that forms the basis of Africa’s future—adolescents.[10] Thus, our findings make an important contribution to the knowledge on the health of African populations under epidemiologic transition. Still, the present study was limited in sample size producing wide confidence intervals of the detected proportions. This calls for independent replications in larger surveys. In addition, we cannot comment on the characteristics of adolescents who did not follow the study invitation, and selection bias might have occurred. Also, the cross-sectional nature of our study bears the problem of recall bias for self-reported diagnoses and symptoms, and the potential of reverse causation for some risk factors. This may limit the interpretability of the associations between infectious diseases and malnutrition. Still, malarial infection, malnutrition and CRFs were objectively measured by well-trained study personnel. For instance, hypertension was defined based on the last two BP measurements performed by a validated, fully-automated device using sex-, age- and height-specific percentiles, to avoid misclassification through investigator-related BP increase (white-coat effect) or conventional BP cut-offs, respectively.

### Conclusions

In conclusion, in this population of rural Ghanaian adolescents, asymptomatic malaria infection, chronic energy deficits and vitamin A deficiency still constitute major health threats. Already at this young age, obesity and hypertension evolve and even co-exist with infectious diseases and nutrient deficits on the individual level. Potential interrelations of malaria, malnutrition, and cardio-metabolic risk factors remain to be investigated for understanding disease trends and ultimately guide resource allocation for health care in sub-Saharan Africa.

## Supporting information

S1 Questionnaires Study questionnaires. (PDF)
