# Supplementary material for: Adolescent health in rural Ghana: A cross-sectional study on the co-occurrence of infectious diseases, malnutrition and cardio-metabolic risk factors
Source: PLoS One. 2017 Jul 20;12(7):e0180436. doi: 10.1371/journal.pone.0180436 (PMC5519039; doi:10.1371/journal.pone.0180436)
Supplement: S1 Questionnaires — (PDF) [file pone.0180436.s001.pdf]

Adolescent health in Agogo, rural Ghana

German Institute of Human Nutrition Potsdam – School of Medical Sciences Kumasi

Informed Consent : ☐ yes ☐ no (**if no, do not proceed**)

Study ID

Name: ______________________________ Date: ______________

Detailed ____

Address: ____

……………….cut here……………….cut here……………….cut here……………….cut here……………….cut here……………….cut here……………….cut here……………….cut here……..

Medical History

Study ID

Age: ……………… (years)

Birth date: _ _ _ _ _ _ _ _

Sex: ☐ male ☐ female

Residence: ☐ Agogo
☐ other:………………………………………………………………………………….

Ethnic group: ☐ Akan
☐ other: ………………………………………………………………………………

Diabetes: ☐ none or not known
☐ known since: ……………………………………………………………………..
Treatment: ☐ none
☐ current antidiabetic drug(s):…………………………
…………………………………………………………………
☐ other:…………………………………………………

Hypertension: ☐ none or not known
☐ known since: ……………………………………………………………………..
Treatment: ☐ none
☐ current anti-hypertension drug(s) …………………
……………………………………………………………..
☐ other:……………………………………………………..

Diabetes known among parents, siblings, children: ☐ no ☐ yes: …………………………………
known among other relatives: ☐ no ☐ yes: …………………………………

Hypertension known among parents, siblings, children: ☐ no ☐ yes: …………………………………
known among other relatives: ☐ no ☐ yes: …………………………………

Did you have <u>fever</u> in the last 2 days? ☐ no ☐ yes

Smoking:

□ never smoked □ quit smoking: when: ………… years: ………… quantity (/day): …………

□ current smoker: years: …………quantity (/day): …………

Other current diseases/conditions:

Disease/condition	Known since	Remarks

Current complaints:

Disease/complaint	No	Yes	Currently (y/n)?	When or since?	Remarks
Fever					
Chills					
Vomiting					
Diarrhoea					
Cough					
Muscle pain					
Dizziness					
Headache, chronic					
Visual problems					
Leg/food ulcer(s)					
Cold feet					
Burning feet					
Increased thrist					
Increased urinating					
Fatigue					
Ravenous appetite					
Itching					
Coma					
Sensory problems					

Current medication:

Drug	Disease	Since	Dose

Previous diseases:

Disease	When?	Remarks

Laboratory Results

Study ID

Fasting glucose: ☐ **mmol/L**

Hemoglobin (Hb): ☐ **g/dL**

Malaria rapid test (MRT), results:

pan-Lactatdehydrogenase (pLDH)
- ☐ **negative**
- ☐ **positive**

HRP2
- ☐ **negative**
- ☐ **positive**

Malaria microscopy, results: ☐ **malaria parasite count (MPS) per 200 white blood cells (WBC)**

Plasmodium species:
- ☐ **P. falciparum**
- ☐ **P. ovale**
- ☐ **P. malariae**
- ☐ **P. vivax**
- ☐ **unknown**

Socioeconomic status

Study ID

Boarding school in:
- ☐ Kumasi
- ☐ Accra
- ☐ Tamale
- ☐ other…………………………………………….

Literacy:
- ☐ able to read well
- ☐ able to read with difficulty
- ☐ unable to read

Education mother:

- ☐ none
- ☐ primary
- ☐ secondary
- ☐ tertiary
- ☐ other: ………………………………………….

Education father:

- ☐ none
- ☐ primary
- ☐ secondary
- ☐ tertiary
- ☐ other: ………………………………………….

Occupation mother:

- ☐ subsistence farmer
- ☐ commercial farmer
- ☐ casual labourer
- ☐ artisan
- ☐ trader
- ☐ businesswoman
- ☐ public servant
- ☐ unemployed
- ☐ other:…………………………………………..

Occupation father:

- ☐ subsistence farmer
- ☐ commercial farmer
- ☐ casual labourer
- ☐ artisan
- ☐ trader
- ☐ businessman
- ☐ public servant
- ☐ unemployed
- ☐ other:…………………………………………

Household:

No. of people living in household:…………………….

No. of siblings:……………………………………….

Did you sleep under a bednet last night? ☐ no ☐ yes

**Household's physical assets:**	Yes	No
Electricity	☐	☐
Pipe-borne water	☐	☐
Radio	☐	☐
Fan	☐	☐
Cupboard	☐	☐
Television	☐	☐
Bicycle	☐	☐
Motor-bike	☐	☐
Fridge	☐	☐
Car/truck/tractor	☐	☐
Cattle	☐	☐

Physical Examination

Study ID

Temp: ____ (ax., °C)

	Systolic BP (mmHg):	Diastolic BP (mmHg):	Heart rate (/min):
**0 minutes**			
**3 minutes**			
**6 minutes**			

Weight: ____ (kg)

Height: ____ (cm)

Waist circumference: ____ (cm)

Hip circumference: ____ (cm)

Mid-upper arm circumference: ____ (cm)
